# Efficacy of different in-office treatments for dentin hypersensitivity: randomized and parallel clinical trial

**DOI:** 10.1590/0103-6440202405487

**Published:** 2024-06-24

**Authors:** Fernanda de Souza e Silva Ramos, André Luiz Fraga Briso, Larissa Albertinazzi, Vitória Marega Marchetti, Marina Trevelin Souza, Ticiane Cestari Fagundes

**Affiliations:** 1 Department of Preventive and Restorative Dentistry, School of Dentistry, São Paulo State University (UNESP), Araçatuba, São Paulo, Brazil.; 2 Vitreous Materials Laboratory, Department of Materials Engineering, Federal University of São Carlos, São Carlos, São Paulo, Brazil.

**Keywords:** Dentin Desensitizing Agents, Randomized Clinical Trial, Clinical Trial, Dentin Sensitivity

## Abstract

The aim of this clinical, prospective, randomized, and parallel study was to evaluate different in-office treatments for dentin hypersensitivity (DH). One hundred ninety-two teeth with non-cavitated root exposures were treated using different desensitizers: fluoride varnish (Duraphat - FLU); bioactive ceramic solution (Biosilicate - BIOS); universal self-etching adhesive (Single Bond Universal - SBU); bioactive photoactivated varnish (PRG filler - SPRG). The degree of DH was analyzed using a visual analog scale (VAS) and computerized visual scale (CoVAS), before treatments and after 7, 15, and 30 days from the first session. Comparisons among desensitizers were performed using the Kruskal-Wallis and Dunn's tests. Friedman test was used to compare between times (p ≤ 0.05). Comparing desensitizers FLU presented a higher value of DH than BIOS using VAS at 7 days, however, no differences were found using CoVAS analysis. Comparing times, BIOS and SBU showed a reduction in DH after 7 days and SBU showed a reduction at 30 days compared to 7 days using VAS. FLU and SPRG groups reduced DH from 15 days to 30 days using VAS. There was a reduction in DH for FLU, BIOS, and SBU after 7 days and for BIOS this reduction also occurred at 30 days when compared to 15 days using CoVAS. SPRG group showed a reduction from 15 to 30 days. All desensitizers tested were able to reduce the initial sensitivity. The bioactive ceramic solution reduced the DH gradually after 30 days using computerized analysis.

## Resumo

O objetivo deste estudo clínico, prospectivo, randomizado e paralelo foi avaliar diferentes tratamentos em consultório para hipersensibilidade dentinária (HD). Cento e noventa e dois dentes com exposições radiculares não cavitadas foram tratados com diferentes dessensibilizantes: verniz fluoretado (Duraphat - FLU); solução cerâmica bioativa (Biosilicato - BIOS); adesivo autocondicionante universal (Single Bond Universal - SBU); verniz fotoativado bioativo (PRG filler - SPRG). O grau de HD foi analisado por meio da escala visual analógica (VAS) e da escala visual computadorizada (CoVAS), antes dos tratamentos e após 7, 15 e 30 dias da primeira sessão. As comparações entre dessensibilizantes foram feitas pelos testes de Kruskal-Wallis e Dunn. O teste de Friedman foi utilizado para comparação entre os tempos (p ≤ 0,05). Comparando os dessensibilizantes, o FLU apresentou um valor de HD maior do que o BIOS usando VAS aos 7 dias, no entanto, nenhuma diferença foi encontrada usando a análise CoVAS. Comparando os tempos, BIOS e SBU apresentaram redução de HD após 7 dias e SBU apresentou redução aos 30 dias em comparação com 7 dias usando VAS. Os grupos FLU e SPRG reduziram a HD em 15 dias a 30 dias usando VAS. Houve redução de HD para FLU, BIOS e SBU após 7 dias e para BIOS essa redução também ocorreu aos 30 dias quando comparada a 15 dias usando CoVAS. O grupo SPRG apresentou redução de 15 para 30 dias. Todos os dessensibilizantes testados foram capazes de reduzir a sensibilidade inicial. A solução de cerâmica bioativa reduziu o HD gradualmente após 30 dias usando análise computadorizada.

## Introduction

Dentin hypersensitivity (DH) is defined as a sharp short pain caused by thermal, tactile, chemical, or osmotic stimuli ^(1,2)^. Among the mechanisms that explain DH, the hydrodynamic theory assumes that external stimuli move the fluid inside the dentin tubules and produce contraction and distension of the odontoblastic processes, stimulating the nerve fibers of the dentin-pulp interface ^2^. This condition is strictly related to root exposure, with or without the presence of non-carious cervical lesions.

Management of DH includes non-invasive treatments for pain relief through occluding dentin tubules and blocking nociceptive transduction/transmission; and restoration or surgical treatments for dental hard and soft tissue defects ^3^.

Currently, the literature presents three categories of products for dentin tubule occlusion: fluoride varnishes, experimental solutions of bioactive products, and products with photocuring agents, as well as the combination of these categories ^3^ ^,^ ^4^ ^,^ ^5^ ^,^ ^6^ ^,^ ^7^. Fluoride varnishes have been most widely used since they have the ability to control the movement of fluids in the dentin tubules through the formation of precipitation of calcium fluoride on the dentin surface ^4^ ^,^ ^5^ ^,^ ^6^ ^,^ ^7^. Experimental solutions of bioactive products include materials that have a structure closer to the mineral portion of teeth ^8^. Solutions with crystalline bioactive ceramics have been able to induce hydroxyl carbonate apatite deposition in open dentinal tubules in vitro ^8^. Bioactive ceramic solution (Biosilicate mixed with distilled water), applied once a week by a dentist, demonstrated the best clinical performance and provided the fastest treatment to reduce DH pain when compared to other dentin desensitizing agents, providing a new option for treating DH ^9^.

Considering the category of products with photocuring agents, universal adhesive has been a treatment option by promoting the sealing of the dentin tubules and the formation of a hybrid layer, neutralizing the hydrodynamic mechanism of DH ^10^. Furthermore, a light-curing varnish was developed containing a resin matrix and inorganic surface pre-reacted glass (SPRG) that allows the release of fluoride (F^−^). This bioactive technology has multifunctional glass particles trapped in the polyacid matrix, which also release other ions such as strontium. (Sr_2_^+^), borato (BO_3_^3−^), aluminum (Al_3_^+^), silicate (SiO_3_^2−^), and sodium (Na^+^). Thus, there is neutralization of acids from food and tissue remineralization, combined with dentin tubules obliteration by means of photocuring monomers ^11^.

Marto et al. ^12^ through a meta-analysis that evaluated randomized clinical trials with different treatments for DH, concluded that only in-office treatments are effective in immediately reducing DH, and may maintain their effectiveness over time; however, more treatment protocols should be studied.

Therefore, the study aimed to evaluate the influence of in-office different treatments for DH, including experimental solutions and recently launched products. The first null hypothesis tested was that there is no difference in the efficacy of different in-office treatments for DH, regardless of the experimental times analyzed. The second null hypothesis tested was that there is no difference in the reduction of DH over time, for each in-office treatment.

## Materials and methods

### Study design and sampling procedure

**Figure 1:**
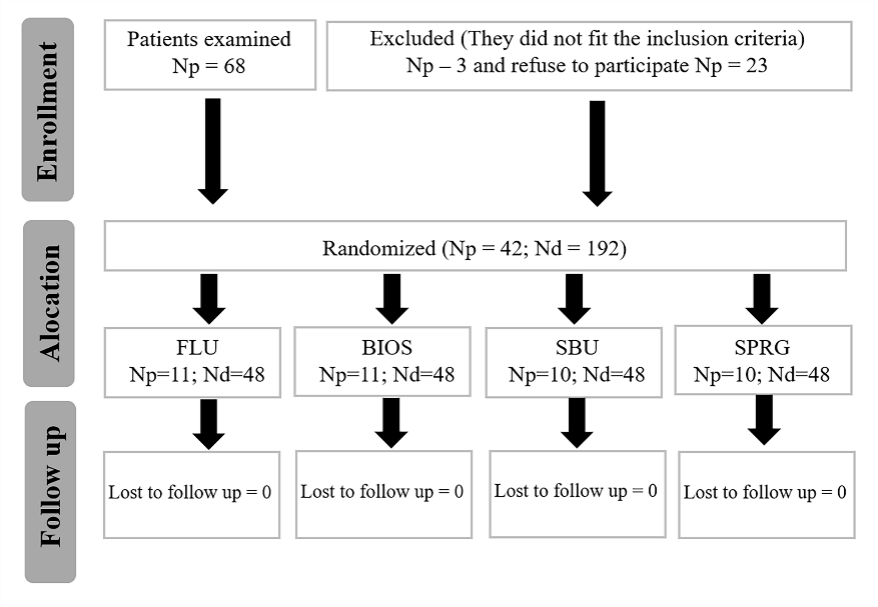
Patient flowchart. Np: patient number, Nd: number of teeth

This research project was approved by the local ethics committee (N° 30122220.1.0000.5420) and registered in the clinical trial database (REBEC: U1111-1251-1091). The study is described as recommended by CONSORT, the flowchart showing the distribution of patients is presented in Figure 1. This is a parallel, prospective, randomized clinical trial that lasted 30 days, from July 2021 to December 2021. Only the volunteers do not know which group they are included in (single-blind protocol).

The calculation of the sample size was performed, based on a previous study ^13^, using the software Sigma Plot 12.0, with an expected difference between means of 0.210 after six months. The parameters used were: significance level (α) = 0.05; test power (1-β) = 0.80; dropout (β)=0.2, resulting in a final sample number of 48 teeth per group. Patients were selected at the undergraduate clinic of the local Faculty of Dentistry. They should be aged between 20-60 years and have at least 1 root exposure with DH, all inclusion and exclusion criteria are described in Box 1 ^6^ ^,^ ^14^. From a total of 68 examined patients, 42 were selected. One hundred ninety-two teeth with DH in non-cavitated root exposures, regardless of location in the dental arch. The patients were randomly allocated to four groups according to the in-office desensitizers: fluoride varnish (FLU), Biosilicate solution (BIOS), universal adhesive (SBU), and varnish with S-PRG particles (SPRG) (Box 2) ^15^ ^,^ ^16^. The degree of DH was evaluated at baseline (before the treatments), 7, 15, and 30 days after the first session, using visual analog scales (VAS) and computerized visual scales (CoVAS).

**Box 1:**
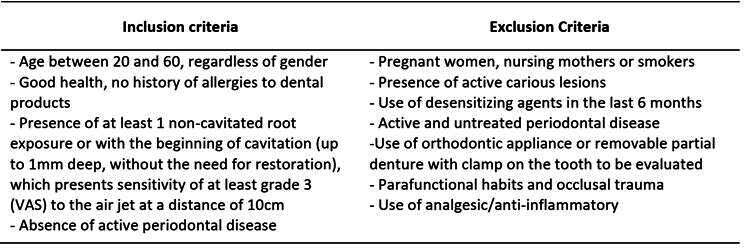
Inclusion and exclusion criteria

### Clinical exams and Randomization

Relative isolation was performed with cotton rolls. The stimuli were performed by means of an air jet, with the triple syringe positioned 1 centimeter from the cervical region, with a duration of 5 s. Immediately, the patient indicates the sensitivity level by the VAS scale consisting of a 10 centimeters horizontal line, the pain is identified by the patient from 1 to 10 points, where 0 means “no pain”, 1-3 “light pain”, 4-6 “moderate pain” and 7-10 “severe pain”(14-17). The patient made a vertical line crossing the horizontal line of the scale to identify the number of intensities of the DH after stimulus application ^14^ ^,^ ^17^. All teeth with DH less than 3 on the VAS scale were excluded.

After being selected, Informed consent was obtained from all subjects. Then, with the aid of a millimeter probe, measurements of the exposure deep and height were performed. The deep of the root exposure could not exceed 1 millimeter. The height of the root exposure was measured considering the distance between the most apical end of the cementoenamel junction and the highest point of the free marginal gingival, no limit was established ^18^.

The height of root exposure and the sensitivity score by the VAS scale were stratified variables in the randomization process. The tooth was considered the sample unit. Eligible teeth were recorded in an Excel spreadsheet according to the height of root exposure and the sensitivity VAS score. In order to have a homogeneous distribution of these two factors in the study groups, the teeth were ordered by the VAS score and divided into two conglomerates: one containing the lowest sensitivity scores and the other with the highest scores. These two clusters were subdivided according to the height of the smallest and major root exposure, resulting in four clusters: (a) lower sensitivity scores (3-7 on the VAS scale) and smaller exposure height (up to 3 millimeters), (b) lower sensitivity scores (3-7 on the VAS scale) and larger exposure height (greater than 3 millimeters), (c) higher sensitivity scores (8-10 on the VAS scale) and smaller exposures height, and (d) higher sensitivity scores (8-10 on the VAS scale) and larger exposures height. This stratified method of randomization was based on another parallel clinical study ^19^.

The conditions of the patient's oral environment included in the study were analyzed through the index of decayed, missing, and filled permanent teeth (DMFT), visible plaque index (VPI), and gum bleeding index (GBI). All information about the patient and teeth was collected to characterize the sample included in this clinical trial.

The CoVAS scale was then used by applying a constant stimulus, by means of an air jet, at a distance of 10 millimeters, on the buccal surface of the dental elements for 30 seconds. During this period, the patient recorded the intensity of discomfort on a scale from 0 to 100 using a manually controlled potentiometer, connected to equipment (Medoc; Ramat Yishai, Northern District, Israel) that records the level of sensitivity felt by the patient during the application of the evaporative stimulus. ^20^ This time of analysis was considered as a baseline.

### Interventions

After the previous evaluations, a buccal retractor was positioned in order to separate the lips and cheek. The tooth that received the desensitizer was isolated with cotton rolls and dried with a jet of air, with the humidity being controlled with a sucker. All protocol steps of each in-office treatment are described in Box 2. The FLU desensitizer was applied in a once-weekly session, for three weeks. The BIOS solution was mixed using 0.15 mg of powder in 1.35 mL of distilled water it was applied in sessions, such as the previous desensitizer ^9^. Light-curing products (SBU and SPRG) were applied and light-cured (LED Radii-cal, SDI Brazil Industry and Commerce LTDA, SP, Brazil) in one session. After interventions, cotton rollers and buccal retractor were removed.

**Box 2:**
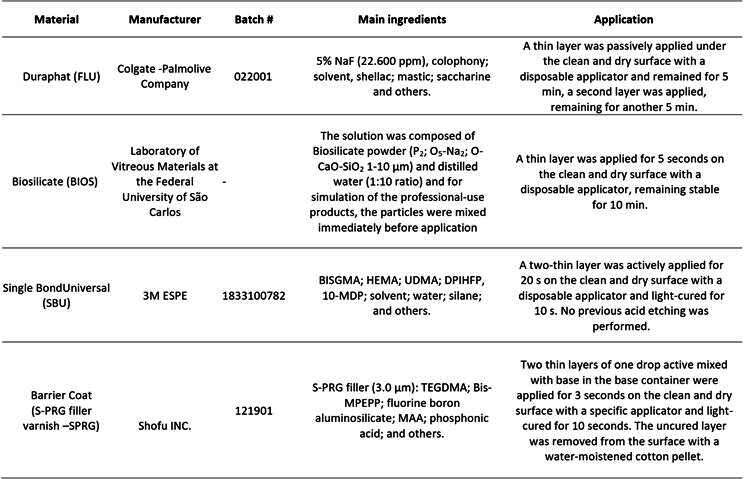
Products to be used in the study and their composition according to the manufacturers.

Abbreviations: TMPnano (nanoparticulate sodium trimetaphosphate) TEGDMA (trimethylene glycol dimethacrylate); BISGMA (diglycidildimethacrylate A); HEMA (Hydroxyethyl methacrylate); UDMA (1,3 glycol dimethacrylate) DPIHFP (Diphenyliodonium hexafluorophosphate); 10-MDP (10-decanediol phosphate methacrylate); Bis-MPEPP (bisphenol A polyethoxy methacrylate); MAA (methacrylic acid)

* According to the manufacturer § Fujimoto, et. al.^26^ Oliveira et al. 2022^27^

### Outcomes

The intensity of DH was measured using the mentioned scales at 7, 15, and 30 days after the first session. In the FLU and BIOS groups, the DH analysis was measured before their reapplication corresponding with each session.

### Statistical analyzes

The results of the deep, height, DMFT, VPI, and GBI of root exposures were presented descriptively. A chi-square statistical test was used to compare differences in proportions of categorical variables between groups. VAS and CoVAS data were evaluated using the Kruskal-Wallis test and Dunn's post-test for comparison between different desensitizers, and the Friedman test was used for comparison between times when the same desensitizer was analyzed. Spearman's correlation test was performed between VAS and CoVAS data. The program used was Jamovi 2.2.5 (Sydney, Australia), with a significance level of 5%.

## Results

In total, 42 patients (15 male and 27 female) with a mean age of 41.5 (±9.53, ranging from 23-58 years) participated in the study. The Mean of DMFT, VPI, and GBI were 11.8 (±6.2), 6.4 (±3.2), and 0.0 (±0) respectively. Root exposures have a mean deep of 0.3 millimeters (±0.26) and height of 1.6 millimeters (±0.9). Descriptive data by group are presented in Table 1.

Data from the VAS scale (Table 2), showed that FLU presented higher DH than BIOS after 7 days from the first session (p≤0.013), with no difference between the other groups. There was no significant difference between the desensitizers when the data were analyzed using CoVAS (Table 3).

**Table 1:** Descriptive demographic data of the study population, with p-value referring to the chi-square test.

Demographics	FLU	BIOS	SBU	SPRG	P value
Age	41.1±7.22	39.9±11.7	41.5±11.1	43.9±8.45	0.213
*Gender*					0.146
Male	1	6	3	4	
Female	10	5	7	6	
Number of patients	11	11	10	10	0.261
DMFT	12.2±5.74	13.3±4.8	10±7.6	11.9±6.97	0.243
VPI	0±0	0±0	0±0	0±0	-
GBI	6.03±3.08	5.26±2.75	6.07±4.24	5.5±1.86	0.254
Height	1.62±0.7	1.68±0.9	1.64±0.9	1.65±0.9	0.213
Depth	0.3±0.3	0.3±0.2	0.3±0.3	0.3±0.2	0.213
*Group of teeth*					0.056
Canine	5	4	1	1	
Central Incisor	2	1	1	0	
Lateral Incisor	2	1	0	1	
Molar	11	15	12	12	
Premolar	30	27	34	34	
*Arch*					0.002
Maxillary	21	29	25	15	
Mandibular	37	19	23	33	

**Table 2:** Median (first and third quartile) of times analyzed and presented by different desensitizing materials according to the VAS scale.

Material	Time			
	*Baseline*	7 days	15 days	30 days
FLU	6(4.3-8)^Aa^	5.5(5-7)^Ba^	5 (3-6)^Ab^	5(3-6)^Ab^
BIOS	5 (3-8)^Aa^	3(2-6)^Ab^	3(2-6)^Ab^	2 (0-6)^Ab^
SBU	7(4.3-8)^Aa^	5(3-7)^ABb^	5(2-6)^Abc^	4.5(1.3-7)^Ac^
SPRG	5(3-7)^Aa^	4(3-6.5)^ABa^	4(2-6.5)^Ab^	3(2-5.5)^Ab^

*The same uppercase letters represent values without significant statistical differences among desensitizing materials for each analysis time. The same lowercase letters represent values without significant statistical differences among analysis times in each desensitizing material.*

**Table 3:** Median (first and third quartile) of times analyzed and presented by different desensitizing materials according to the CoVAS scale

Material	Time			
	*Baseline*	7 days	15 days	30 days
FLU	26(17.8-100)^Aa^	18(15-34.8)^Ab^	21(11.5-28.5)^Ab^	17.5(13-100)^Ab^
BIOS	18(13-100)^Aa^	16(7-100)^Ab^	16(9-100)^Ab^	14(4-100)^Ac^
SBU	24.5(16-100)^Aa^	18(10.5-84.8)^Ab^	16(4-82.3)^Ab^	17(4.3-36.3)^Ab^
SPRG	18(13-30.5)^Aa^	16(12-29)^Aa^	16(9-24.5)^Ab^	14(12-100)^Ab^

*The same uppercase letters represent values without significant statistical differences among desensitizing materials for each analysis time. The same lowercase letters represent values without significant statistical differences among analysis times in each desensitizing material.*

All time analyses were compared considering after the first session, for example: FLU and BIOS received the last analysis 7 days after the third application. Considering VAS data, BIOS and SBU showed a significant reduction in sensitivity after 7 days, remaining after 30 days (p< 0.001); and the SBU showed a significant reduction in the comparison of 30 days with 7 days (p≤0.013). There was a reduction in sensitivity at 15 days, remaining after 30 days for FLU and SPRG (p< 0.001). For the CoVAS analysis, there was a significant reduction inDH in the FLU, BIOS, and SBU at 7 days, remaining at 15 and 30 days (p< 0.001), and BIOS showed a significant reduction at 30 days compared to 15 days. In the SPRG group, a significant reduction was found from 15 days remaining at 30 days (p< 0.001).

Statistical analysis showed a positive correlation between VAS and CoVAS, regardless of the analysis time (Table 4), indicating that they have a direct correlation.

**Table 4:** Spearman correlation coefficient (ƿ) and test p-value comparing VAS and CoVAS analyses.

Correlation	*Time*			
	*Baseline*	7 days	15 days	30 days
Coefficient (ƿ)	0.393	0.389	0.394	0.424
p-value	< .001	< .001	< .001	< .001

## Discussion

The literature demonstrates that DH influences the life quality of patients, affecting daily activities such as talking, drinking, eating, and brushing teeth ^1^ ^,^ ^12^. Etiological factors associated with the development of DH are soft tissue dehiscence, including aggressive brushing, which can cause apical displacement of the gingival margins thereby leading to exposure of root dentin ^21^. The conditions of the patient's oral environment in this study, corroborate with these aspects since the results of GBI equal to zero may be associated with an aggressive brushing, also evidenced by the low VPI. Important factors are the size of the lesions and the age of the patients, as according to West et al. ^22^ small newly exposed dentin lesions, exhibiting minimal tooth wear at the cementoenamel junction, may cause intensified sensitivity in young adult individuals. In the present study, approximate dimensions of 1.6 mm in height, 0.3 mm in depth, and a mean age of 41 years were observed, which may contribute to the sensitivity shown by patients ^22^. The VAS and CoVAS analyses used in this study were chosen because of their easy application and good patient tolerance. ^20^ Time-consuming analysis can cause patient stress and inaccurate data ^20^. Another factor that is important to highlight is that DH is a condition that is difficult to quantify due to subjectivity ^7^. In this study, it was decided that evaporative stimuli with air jets were used to quantify DH, as it has been shown to be more accurate than the tactile method ^7^.

In the present study, when the desensitizers were compared, FLU showed a significantly greater DH than other desensitizers in 7 days using the VAS scale, thus refusing the first null hypothesis. This is probably due to the fact that this material releases a greater amount of fluoride in the first two weeks, which demonstrates a significant reduction in DH after 15 days, with no difference when compared to other materials ^6^. This result, as well as the result of the CoVAS scale, corroborates with previous studies, which demonstrate that the NaF-based varnish has great sensitivity relief in the first weeks of treatment ^6^ ^,^ ^7^. The study by Ritter et al. evaluated the application of two NaF-based varnishes and demonstrated significantly lower sensitivity values on the VAS scale even after 24 weeks of follow-up ^6^. However, other clinical studies demonstrated a reduction in DH up to the first month of treatment, which is lost after three months of application ^7^. CaF_2_ precipitates produced by applying NaF varnish are not resistant to the oral environment conditions, and fluoride can be released into saliva over time ^7^.

The second null hypothesis of this clinical trial was also rejected, as all evaluated desensitizers showed a significant reduction in DH between times. The BIOS showed this reduction from the 7-day, remaining until 30 days for the VAS analysis and showing a significant reduction in this time for the CoVAS analysis. A study with scanning electron microscopy demonstrated a considerable occlusion of the dentin tubules by Biosilicate ^8^ ^,^ ^16^. Another factor that explains this result is the solid character of these fine particles, which accelerate the reaction of this material on the dentin surface, which helps to initiate the obliteration action by mechanical fixation and by the formation of a barrier composed of hydroxy carbonate apatite ^8^ ^,^ ^9^. In a clinical study, there was also a significant reduction in DH when different vehicles for the BIOS were tested, with better results being obtained when in a distilled water solution during the first 30 days, corroborating our study ^9^.

Regarding the SBU, a reduction in DH at 7 days is demonstrated, which was maintained over time in both analyses performed. This result corroborates with previous clinical studies that demonstrated a reduction in DH with the use of a universal adhesive ^10^ ^,^ ^23^. This is due to the acidic monomers present in this type of adhesive, which promotes the simultaneous dissolution of the smear layer and creates a hybrid layer without exposing the collagen fibrils, reducing the risk of collapse of the collagen network and promoting a desensitizing effect ^10^ ^,^ ^23^. This fact can be attributed to the specific carboxylate and/or phosphate monomers that can ionically bind to Ca_2_^+^ in hydroxyapatite, in addition to SBU, it also has the phosphate acid monomer, 10-MDP (10-methacryloyloxydecyl dihydrogen phosphate), which chemically binds to hydroxyapatite, forming hydrolytically stable calcium salts in the form of “nano-layering” ^24^. This chemical adhesion improves the mechanical and structural stability of collagen, leading to a stable dentin matrix after resin monomer infiltration and decreases hydraulic conductance ^10^ ^,^ ^24^. Askari et al. ^10^, compared the effectiveness of the universal adhesive with propolis extract-based desensitizers, where the SBU was able to reduce DH by 81.5% after 1 day of evaluation. It was observed that in the present study, there was a similar reduction (72.3%) after 7 days of follow-up. A slight decrease in the effectiveness of this desensitizer occurred after 15 days and remained after 30 days, such as in other clinical studies cited above ^10^.

The SPRG also showed a reduction in DH in both analyses performed after the 15 days. Ravishankar et al. also found these results, when compared this material to two other desensitizing agents, concluding that all were effective in reducing DH after 30 days ^11^. However, this clinical study using SPRG evaluated only 20 teeth per group, making it difficult to compare with our results ^11^. Materials containing the S-PRG particle are relatively new in the dental market, with few studies of them. Therefore, some in vitro studies have demonstrated that six ions (Na^+^, F^−^, Al_3_^+^, BO_3_^3−^, Sr_2_^+^, and SiO_3_^2−^) are released by the SPRG molecule, having buffering capacity, which contributes to inhibiting demineralization of the tooth surface ^15^. The Al_3_^+^ ion is associated with a greater release of F^-^ ion whose association with Na^+^ ion forms a soluble fluoride salt that can contribute to a rapid release of fluorine into the buccal medium. Furthermore, SiO_3_^2−^ ion acts as a nucleation center for the formation of calcium phosphate on the dentin ^15^. In vitro studies have demonstrated that SPRG desensitizer is capable of controlling the permeability of human dentin under erosive conditions by mineral deposits on dentin ^16^ ^,^ ^25^. This ion release and consequent deposition may explain the significant reduction in DH after 15 days, remaining after 30 days observed in this present study.

It is important to emphasize that two pain sensitivity quantification scales were (VAS and CoVAS) which showed a positive correlation, corroborating with the study of Briso et al. ^20^ Furthermore, the present study presents some limitations such as the difficulty in select patients, due to the restrictions imposed by the COVID-19 pandemic, which explains the long recruitment time. Another limitation is the short follow-up of 30 days for the first section, thus, longer studies that assess the durability of these desensitizers, as well as the need for their reapplication, can be performed. In addition, studies that evaluate the mechanism of action, the morphological alterations of the dentin, and the permeability alterations after the application of this in-office treatment are necessary, mainly the BIOS and the SPRG which are still little explored in the literature.

Considering the limitations of this study, it can be concluded that the desensitizing products evaluated were able to reduce the initial sensitivity. The bioactive ceramic solution reduced the dentin hypersensitivity gradually after 30 days using computerized analysis.
